# Higher order chemical reaction effects on $\text {Cu}{-}\text {H}_2\text {O}$ nanofluid flow over a vertical plate

**DOI:** 10.1038/s41598-022-20155-1

**Published:** 2022-10-11

**Authors:** Padmaja K, Rushi Kumar B

**Affiliations:** Department of Mathematics, School of Advanced Sciences, VIT, Vellore, Tamil Nadu 632014 India

**Keywords:** Applied mathematics, Chemical engineering, Mechanical engineering

## Abstract

Many fluids used in industries will possess a uniform velocity acting along with it. Although a few researchers have analyzed the fluid flow along with a constant velocity but such modeling in nanofluids is quite new. The novelty of this work is the numerical evaluation of a nanofluid with a constant velocity through a vertical plate in a porous medium under Dufour as well as Soret impacts coupled with a higher order chemical reaction. A rotating MHD nanofluid is investigated for both heat as well as mass transfer. An incompressible, steady-state fluid is subjected to flow through a semi-infinite plate by taking into account viscous dissipation as well as a magnetic field. Flow equations are typically represented by PDEs that are nonlinear and coupled. The PDEs are changed to ODEs by similarity transformation variables. Runge-Kutta method of $4{\text {th}}$ order accuracy along with shooting technique is employed to solve the converted system of ODEs. $\text {Cu}{-}\text {H}_2\text {O}$ is used to provide an in-depth analysis of the examined problem. In order to account for practical considerations, the maximum order of the chemical reaction is limited to 3 and a comparative analysis is provided for $1{\text {st}}$ and $3{\text {rd}}$ order chemical reactions. For different physical quantities, different numerical values that are obtained using MATLAB are used to analyze various properties regarding the flow. Heat transfer, and mass transfer rates are discussed using graphs and tables. Compared to low order chemical reactions, high order chemical reactions allow higher rates at which the reaction takes place, thus allowing greater rates of heat and mass transfer.

## Introduction

There is a wide range of applications for heat, and mass transfer with higher order chemical reactions through porous media in chemical and water industries. Industrial processes like filtration, distillation, cooling, and drying rely on heat, and mass transfer, in conjunction with chemical reactions. In industries, the rate at which a process occurs is extremely essential; the faster the rate, the lesser time is required in the process, which in turn reduces the amount of time required for storage, resulting in a process that is more efficient and comparatively affordable. The order of a chemical reaction explains the relationship between the concentration of a species and the rate at which the reaction takes place. A reaction is of $n\text {th}$ order if the rate of a reaction is directly proportional to the *nth* power of concentration of the species. Chemical reactions occur at a faster rate as the order of the reaction increases. It is necessary to have faster rates of chemical reaction in order to manufacture chemicals such as fertilizers, polymers, and color dyes on a large scale. The effects of higher order chemical reactions in porous media have previously been studied by Rahman et al.^1^, Mallikarjuna et al.^2^, Rajani et al.^3^, Matta et al.^4^ and Sastry et al.^5^.

Thermal energy is used extensively in industrial operations including expulsion, papermaking, and cooling computer chips to generate finished products with desired qualities. A significant role for viscous dissipation, and joule heating is to modify temperatures by acting as an energy source. Heat transfer rates change when temperatures do. Several authors Chen^6^, Alam et al.^7^, Jaber^8^, Pandey and Kumar^9^, Prakash et al.^10^, Reddy et al.^11^ have researched this topic extensively.

By passing a fluid through the product, heat can be either transferred away from or transferred towards the product. There must be ways of transferring heat efficiently without wasting significant energy. Some metals, which have high thermal conductivity, can be combined with a liquid, such as water, to transfer heat efficiently. In their research, Choi and Eastman^12^ used nanometer-sized particles and a base fluid to develop nanofluids, which are colloidal suspensions containing nanometer-sized particles. As a result of its high compatibility, this fluid is capable of inducing or reducing heat transfer. The study of nanofluids to enhance the rates of heat transfer in solar thermal applications has been done by Sheikholeslami^13–15^.

In their study, Jagadha et al.^16^ conducted an investigation on the flow of nanofluid over porous vertical plate taking into account factors like dispersion, radiation, dissipation, chemical reaction, as well as Brownian motion, and some dimensionless number effects. Swain et al.^17^ made an analysis of the effect of viscous dissipation, joule heating, magnetic parameter, and suction parameter on the MHD flow of a nanofluid with a higher order chemical reaction. This study was conducted through a stretching surface in a permeable medium.

In an analytical study, Alaidrous and Eid^18^ examined how nanofluid moves through a porous stretching sheet by considering the impacts of higher order chemical reaction, radiation, Joule heating, and viscous dissipation. Gopal et al.^19^ investigated numerically how an MHD nanofluid flow in a porous stretching sheet is affected by the impacts of higher order chemical reaction as well as viscous dissipation.

With the help of Water and Ethylene Glycol mixture as the base fluid and $\text {Ag}{-}\text {TiO}_2/WEG$ Casson hybrid nanoparticles Krishna et al.^20^ analysed the flow of a MHD Casson hybrid nanofluid flow over an infinitely exponential accelerated vertical porous surface. An investigation of the unsteady MHD $\text {Al}_2\text {O}_3, \text {TiO}_2$ nanofluid flow over a moving vertical porous surface with a uniform transverse magnetic field and heat radiation, absorption effects has been done by Krishna et al.^21^.

Based on the aforementioned research and its conceivable relevance to a number of scientific disciplines, it would be worthwhile to consider and investigate the aspects of higher order chemical reaction, Soret as well as Dufour effects on $\text {Cu}{-}\text {H}_2\text {O}$ nanofluid in a vertical plate contained in a porous medium concurrently with viscous dissipation, magnetic effects. Consideration is given to copper nanoparticles (Cu) due to their widespread use in food processing, water purification, and chemical processing (Dankovick and Smith^22^).

Many fluids used in industries will possess a uniform velocity acting along with it. Although a few researchers^2,7^ have analyzed the fluid flow along with a constant velocity but such modeling in nanofluids is quite new. The originality of this work is in the numerical evaluation of a nanofluid travelling at a constant velocity through a vertical plate in porous media while being subjected to Dufour and Soret impacts in conjunction with a higher-order chemical reaction. We analyze the boundary layer equations by incorporating the nanoparticles and the base fluid’s thermodynamical properties along with a uniform velocity. Through illustrations of the obtained graphs, we explore how each parameter affects velocity, temperature, and concentration. Using tables, we examine local skin friction coefficient, Nusselt number and Sherwood pertaining to fluid flow parameters.

## Mathematical modeling

**Figure 1:**
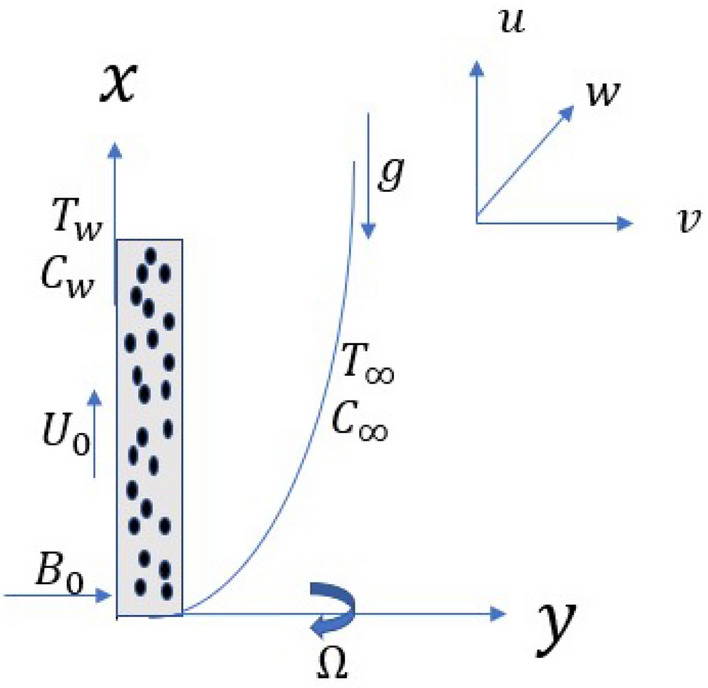
Flow geometry.

A vertical, semi-infinite plate contained in a porous medium is oriented along the *x*-axis. Envision a steady state nanofluid flow of a uniform velocity $U_0$ in the *x*-axis and the *y*-axis is normal to the *x*-axis as shown in Fig. 1. Nanofluid considered is incompressible, laminar and at steady state rotating along the *y*-axis with velocity $\Omega$. A uniform magnetic field $B_o$ is taken to be acting along the *y*-axis which is assumed to be electrically non conducting. We assume that the magnetic Reynolds number of the flow is taken to be small enough so that the induced magnetic field is negligible in comparison with applied one, so that the magnetic field acts along *y*-axis. Nanofluid provides an environment for chemical reactions of order *n* among species diffusing in it. Species concentrations near the wall and in the free stream significantly affect the flow. Because of this, we take into account the Dufour and Soret effects. Buoyancy is produced by a temperature disparity between the fluid and its surroundings. In this case, we take into account the buoyancy effects of both temperature and concentration. On the basis of aforementioned assumptions and the Boussinesq approximation, we can deduce these equations:

1 $$\begin{aligned}{}&\frac{\partial u}{\partial x}+\frac{\partial v}{\partial y}\,=\,0, \end{aligned}$$

2 $$\begin{aligned}{}&\rho _{nf}(u\frac{\partial u}{\partial x}+v\frac{\partial u}{\partial y})\,=\,\mu _{nf}(\frac{\partial ^2 u}{\partial y^2 })-\frac{\mu _{nf}}{K^*}(U_0-u)+2\Omega \rho _{nf}w +\sigma _{nf} B_o^2(U_0-u)+(\rho \beta )_{nf}g(T-T_\infty )+(\rho \beta )_{nf}g(C-C_\infty ), \end{aligned}$$

3 $$\begin{aligned}{}&\rho _{nf}(u\frac{\partial w}{\partial x}+v\frac{\partial w}{\partial y})\,=\,\mu _{nf}(\frac{\partial ^2 w}{\partial y^2 })-\frac{\mu _{nf}}{K^*}w+2\Omega \rho _{nf}(U_0-u)-\sigma _{nf} B_o^2w, \end{aligned}$$

4 $$\begin{aligned}{}&u\frac{\partial T}{\partial x}+v\frac{\partial T}{\partial y}\,=\,\frac{\kappa _{nf}}{(\rho C_p)_{nf}}\frac{\partial ^2 T}{\partial y^2}+\frac{D_m K_T}{C_sC_p}\frac{\partial ^2 C}{\partial y^2}+\frac{\mu _{nf}}{(\rho C_p)_{nf}}[(\frac{\partial u}{\partial x})^2+(\frac{\partial w}{\partial y})^2] +\frac{\sigma _{nf} B_o^2}{(\rho C_p)_{nf}}[(U_0-u)^2+w^2], \end{aligned}$$

5 $$\begin{aligned}{}&u\frac{\partial C}{\partial x}+v\frac{\partial C}{\partial y}\,=\,D_m\frac{\partial ^2 C}{\partial y^2}+\frac{D_m K_T}{T_m}\frac{\partial ^2 T}{\partial y^2}-k_r(C-C_\infty )^n. \end{aligned}$$

With boundary conditions,

6 $$\begin{aligned} u\,=\, & {} 0, v\,=\, v_0(x), w\,=\, 0, T\,=\, T_w, C\,=\, C_w \quad \text {at}\quad y\,=\,0 \nonumber \\&u \rightarrow U_0, w \rightarrow 0, T \rightarrow T_{\infty }, C \rightarrow C_{\infty } \quad \text {as} \quad y \rightarrow \infty . \end{aligned}$$

Here, *u*, *v*, *w* are velocities in *x*, *y*, *z* directions. $B_0$ is applied magnetic field, $C_p$ is specific heat at constant pressure, $C_s$ is concentration susceptibility, *C* is nanofluid’s local concentration, $C_w$ is nanofluid’s concentration on wall, $C_\infty$ is nanofluid’s concentration in free stream, $D_m$ is molecular diffusivity, *g* denotes acceleration due to gravity, $K^*$ denotes permeability parameter, $K_T$ denotes thermal diffusivity ratio, $k_r$ denotes chemical reaction parameter, *n* is chemical reaction’s order, *T* is nanofluid’s local temperature, $T_w$ is nanofluid’s temperature on the wall, $T_\infty$ is nanofluid’s temperature in free stream, $T_m$ is mean fluid temperature.

We now have two velocities $U_0$ (a constant velocity acting along with the fluid) and *u* (velocity along the x-axis). For the ease of solving the problem, we define a velocity $u_1$, $u_1$ = $U_0-u$ as per the idea used by Raptis and Pfrdikis^23^.

As a consequence of applying the transformation $u_1$ = $U_0-u$, the Eqs. (1 to 5) and the boundary condition (6) are transformed into the following equations.

7 $$\begin{aligned}{}&-\frac{\partial u_1}{\partial x}+\frac{\partial v}{\partial y}\,=\,0, \end{aligned}$$

8 $$\begin{aligned}{}&\rho _{nf}((U_0-u_1)\frac{\partial u_1}{\partial x}+v\frac{\partial u_1}{\partial y})\,=\,\mu _{nf}(\frac{\partial ^2 u_1}{\partial y^2 })-\frac{\mu _{nf}}{K^*}u_1-2\Omega \rho _{nf}w-\sigma _{nf} B_o^2u_1-(\rho \beta )_{nf}g(T-T_\infty )-(\rho \beta )_{nf}g(C-C_\infty ), \end{aligned}$$

9 $$\begin{aligned}{}&\rho _{nf}((U_0-u_1)\frac{\partial w}{\partial x}+v\frac{\partial w}{\partial y})\,=\,\mu _{nf}(\frac{\partial ^2 w}{\partial y^2 })-\frac{\mu _{nf}}{K^*}w+2\Omega \rho _{nf}u_1-\sigma _{nf} B_o^2w, \end{aligned}$$

10 $$\begin{aligned}{}&(U_0-u_1)\frac{\partial T}{\partial x}+v\frac{\partial T}{\partial y}\,=\,\frac{\kappa _{nf}}{(\rho C_p)_{nf}}\frac{\partial ^2 T}{\partial y^2}+\frac{D_m K_T}{C_sC_p}\frac{\partial ^2 C}{\partial y^2}+\frac{\mu _{nf}}{(\rho C_p)_{nf}}[(\frac{\partial u_1}{\partial x})^2+(\frac{\partial w}{\partial y})^2] +{\frac{\sigma _{nf} B_o^2}{(\rho C_p)_{nf}}[(u_1)^2+w^2]}, \end{aligned}$$

11 $$\begin{aligned}{}&(U_0-u_1)\frac{\partial C}{\partial x}+v\frac{\partial C}{\partial y}\,=\,D_m\frac{\partial ^2 C}{\partial y^2}+\frac{D_m K_T}{T_m}\frac{\partial ^2 T}{\partial y^2}-k_r(C-C_\infty )^n. \end{aligned}$$

The boundary conditions are

12 $$\begin{aligned} u_1\,=\, & {} U_0, v\,=\, v_0(x), w\,=\, 0, T\,=\, T_w, C\,=\, C_w \quad \text {at}\quad y\,=\,0 \nonumber \\&u_1 \rightarrow 0, w \rightarrow 0, T \rightarrow T_{\infty }, C \rightarrow C_{\infty } \quad \text {as} \quad y \rightarrow \infty . \end{aligned}$$

Thermodynamical properties of nanofluids are

13 $$\begin{aligned} \begin{matrix} \mu _{nf}\,=\,\frac{\mu _f}{(1-\phi _{0} )^{2.5}},\nu _{nf}\,=\,\frac{\mu _{nf}}{\rho _{nf}}, \rho _{nf}\,=\,(1-\phi _{0} )\rho _f+\phi _{0} \rho _s, (\rho \beta )_{nf}\,=\,(1-\phi _{0} )(\rho \beta )_f+\phi _{0} (\rho \beta )_s , \\ (\rho C_p)_{nf}\,=\,(1-\phi _{0} )(\rho C_p)_f+\phi _{0} (\rho C_p)_s, \kappa _{nf}\,=\,\frac{(\kappa _s+2\kappa _f)-2\phi _{0}(\kappa _f-\kappa _s)}{(\kappa _s+2\kappa _f)+2\phi _{0} (\kappa _f-\kappa _s)}. \end{matrix} \end{aligned}$$

Here, $\beta _{f}$ is base fluid’s thermal expansion coefficient, $\beta _{nf}$ is nanofluid’s thermal expansion coefficient, $\beta _{s}$ is nanoparticle’s thermal expansion coefficient, $\rho _{f}$ is base fluid’s density, $\rho _{s}$ is nanoparticle’s density, $\rho _{nf}$ is nanofluid’s density, $(\rho C_p)_{nf}$ is nanofluid’s heat capacitance, $\mu _{f}$ is base fluid’s dynamic viscosity, $\mu _{s}$ is nanoparticle’s dynamic viscosity, $\mu _{nf}$ is nanofluid’s dynamic viscosity, $\nu _{f}$ is base fluid’s kinematic viscosity, $\nu _{s}$ is kinematic viscosity of nanoparticles, $\sigma _{nf}$ is nanofluid’s electrical conductivity, $\nu _{nf}$ is nanofluid’s kinematic viscosity, $\phi _{0}$ is nanofluid’s volume fraction, $\kappa _{nf}$ is nanofluid’s conductivity, $(C_p)_{nf}$ is nanofluid’s specific heat.

The Eqs. (7)–(11) serve as the basic governing equations here. Utilizing transformations (14), the governing Eqs. (7)–(11), as well as the boundary conditions (12) are solved.

14 $$\begin{aligned} \begin{aligned} \eta \,=\,y \sqrt{\frac{U_0}{{2\nu x}}}, g(\eta )\,=\,\frac{w}{U_0}, \theta \,=\,\frac{T-T_\infty }{T_w-T\infty }, \phi \,=\,\frac{C-C_\infty }{C_w-C_\infty }, \psi \,=\,\sqrt{2\nu xU_0}f(\eta ),\\ u_1\,=\,\frac{\partial \psi }{\partial y}\,=\,U_0f'(\eta ) \Rightarrow \frac{u}{U_0}\,=\,1-f'(\eta ), f_w\,=\,v_0(x)\sqrt{\frac{2x}{U_0 \nu } }. \end{aligned} \end{aligned}$$

By substituting (13) and (14) in Eqs. (7)–(11),

15 $$\begin{aligned}{}&v\,=\,-\sqrt{\frac{U_0\nu }{2x}}(\eta f'-f), \end{aligned}$$

16 $$\begin{aligned}{}&f'''\,=\,\frac{1}{a_1} [-f''(\eta -f)+\frac{a_1f'}{K}+Rg+\frac{M f'}{a_2}+\frac{a_3}{a_2}Ri(\theta +N \phi )], \end{aligned}$$

17 $$\begin{aligned}{}&g''\,=\,\frac{1}{a_1} [ -g'(\eta -f)+\frac{a_1g}{K}-Rf'+a_4Mg], \end{aligned}$$

18 $$\begin{aligned}{}&\theta ''\,=\,\frac{1}{a_5-a_6PrScSoDu} [a_6(\eta -f)(DuScPr\phi '-\theta ')-MEcPr[(f')^2+g^2]-a_6a_7PrEc[(f'')^2+(g')^2]+a_6PrDuSc\gamma \phi ^n], \end{aligned}$$

19 $$\begin{aligned}\phi^{\prime\prime}\,&=\,\frac{1}{a_5-a_6PrScSoDu} [Sc(\eta -f)(a_6SoPr\theta^{\prime}-a_5\phi^{\prime})-MEcPrScSo[(f^\prime)^2+g^2]\\ &\quad-a_6a_7PrEcScSo[(f^{\prime\prime})^2+(g^{\prime})^2] +a_5Sc\gamma \phi ^n]. \end{aligned}$$

The associated boundary conditions are

20 $$\begin{gathered} f = \, f_{w} ,f^{\prime}\, = \, 1,g\, = \, 0,\theta \, = \, 1,\phi \, = \, 1\quad {\text{at}}\quad \eta \, = \, 0 \hfill \\ f^{\prime} \to 0,g \to 0,\theta \to 0,\phi \to 0\quad {\text{as}}\quad \eta \to \infty , \hfill \\ \end{gathered}$$

where primes refer to derivatives about $\eta$; Here

21 $$\begin{aligned} & M\,=\,\frac{2\sigma _{nf} B_o^2x}{U_0\rho _f } , K\,=\,\frac{2\nu _fx}{K^*U_0}, Ri\,=\,\frac{(\nu _f )^2 g\beta _f(T_w-T_\infty )}{x(U_0)^2}, N\,=\,\frac{T_w-T_\infty }{C_w-C_\infty }, {Re}_x\,=\,\frac{xU_0}{\nu },\\& R\,=\,\frac{4x\Omega }{U_0}, Pr\,=\,\frac{(\mu C_p)_f}{K_f}, Du\,=\,\frac{D_mK_t}{C_sC_p\nu _f }\frac{C_w-C_\infty }{T_w-T_\infty }, \gamma \,=\,\frac{2k_rx}{U_0} (C_w-C_\infty )^{n-1}, \\& Sc\,=\,\frac{\nu _f }{D_m}, So\,=\,\frac{D_mK_t}{\nu _fT_m }\frac{T_w-T\infty }{C_w-C_\infty }, Ec\,=\,\frac{{U_0}^2}{C_p(T_w-T_\infty )}, a_1\,=\,\frac{1}{(1-{\phi }_{0} )^{2.5}}\frac{1}{(1-\phi _{0} )+\phi _{0} (\frac{\rho _s}{\rho _f})}, \\& a_2\,=\,(1-\phi _{0} )+\phi _{0} (\frac{\rho _s}{\rho _f}), a_3\,=\,(1-\phi _{0} )+\phi _{0} (\frac{(\rho \beta )_s}{(\rho \beta )_f}),\\& a_4\,=\,\frac{1}{(1-\phi _{0} )+\phi _{0} (\frac{\rho _s}{\rho _f})}, a_5\,=\, \frac{(\kappa _s+\kappa _f)-2\phi _0(\kappa _f-\kappa _s)}{(\kappa _s+\kappa _f)+2\phi _0 (\kappa _f+\kappa _s)}.\frac{1}{\kappa _f}, \\& a_6\,=\,((1-\phi _{0} )(\rho C_p)_f+\phi _{0}(\rho C_p)_s ), a_7\,=\,\frac{(1-{\phi }_{0} )^{2.5}}{((1-\phi _{0} )(\rho C_p)_f+\phi _{0}(\rho C_p)_s )}. \end{aligned}$$

In the above quantities, $T_w$ denotes temperature on wall and $C_w$ is concentration on wall; $\theta$ and $\phi$ are dimensionless temperature and dimensionless concentration; *Ec* is Eckert number; *M* symbolizes dimensionless magnetic field parameter; *K* is dimensionless permeability parameter; $\gamma$ symbolizes dimensionless chemical reaction parameter; $Re_x$ is Reynolds number; *R* is rotation parameter; *Ri* symbolizes Richardson number; *Pr* symbolizes Prandtl number; *Sc* symbolizes Schmidt number; *Du* and *So* symbolize Dufour and Soret effects. For practical applications, local skin friction coefficient, Nusselt number, Sherwood number are relevant physical quantities, that are defined as:

- Local Skin friction coefficient, $Cf_x\,=\,\frac{{\tau }_w}{{\rho }_f {v_0}^2}\Rightarrow \sqrt{2} Cf_x(1-\phi _{0})^{2.5}\,=\,f''(0)$.
- Local Nusselt number, $Nu_x\,=\,\frac{xq_w}{K_f(T_w-T_{\infty })} \Rightarrow \sqrt{2} \frac{Nu_x}{Re_x} \frac{K_f}{K_{nf}}\,=\,-\theta '(0).$
- Local Sherwood number, $Sh_x\,=\,\frac{xq_m}{D_B(C_w-C_{\infty })} \Rightarrow \sqrt{2} \frac{Sh_x}{Re_x} \,=\,-\phi '(0).$

where ${\tau }_w\,=\,{\mu }_{nf}(\frac{\partial u}{\partial y})_{y\,=\,0}$, is the wall shear stress, ${q}_w\,=\,-{K}_{nf}(\frac{\partial T}{\partial y})_{y\,=\,0}$, is the wall heat flux, ${q}_m\,=\,-{D}_{B}(\frac{\partial C}{\partial y})_{y\,=\,0}$, is the wall mass flux.

## Method of solution

In order to solve Eqs. (16)–(20) using the bvp4c package in MATLAB, the construction of functions that give solutions to differential equations with boundary conditions is necessary. Consider, $f \,=\, f(1); f' \,=\, f(2); f'' \,=\, f(3); g \,=\, f(4); g' \,=\, f(5); \theta \,=\, f(6); \theta ' \,=\, f(7); \phi \,=\, f(8); \phi '\,=\,f(9);$

Eqs. (16)–(19) are transformed into the following first order differential equations.

$$\begin{aligned} \begin{bmatrix} f' \\ f'' \\ f'''\\ g' \\ g'' \\ \theta ' \\ \theta '' \\ \phi ' \\ \phi '' \end{bmatrix} \,=\, \begin{bmatrix} f(2) \\ f(3) \\ \frac{1}{a_1}((f(1)f(3))+(\frac{a_1f(2)}{K})+(\frac{M}{a_2}f(2))-(\eta f(3))+(Rf(4)))+(\frac{a_3}{a_2}Ri((f(6)))+Nf(8))) \\ f(5) \\ \frac{1}{a_1}((f(1)f(4))-(Rf(2))+(\frac{a_1f(4)}{K})+(a_4Mf(4))-(\eta f(4)) \\ f(7) \\ \frac{1}{a_5-a_6PrScSoDu}[a_6(\eta -f(1))(DuScPrf(9)-f(7))-MEcPr[(f(2))^2+(f(4))^2]- {a_6a_7PrEc}[(f(3))^2+(f(5))^2]+a_6PrDuSc\gamma (f(8))^n] \\ f(9) \\ \frac{1}{a_5-a_6PrScSoDu} [Sc(\eta -f(1))(a_6SoPrf(7)-f(9))-MEcPrScSo[(f(2))^2+(f(4)^2]-a_6a_7PrEcScSo[(f(3))^2+(f(5))^2]+a_5Sc\gamma (f(8))^n]. \end{bmatrix} \end{aligned}$$

When this problem is viewed as an initial value problem, a numerical solution can be found. MATLAB’s bvp4c package has been used to solve these equations.

## Results and discussion

**Figure 2:**
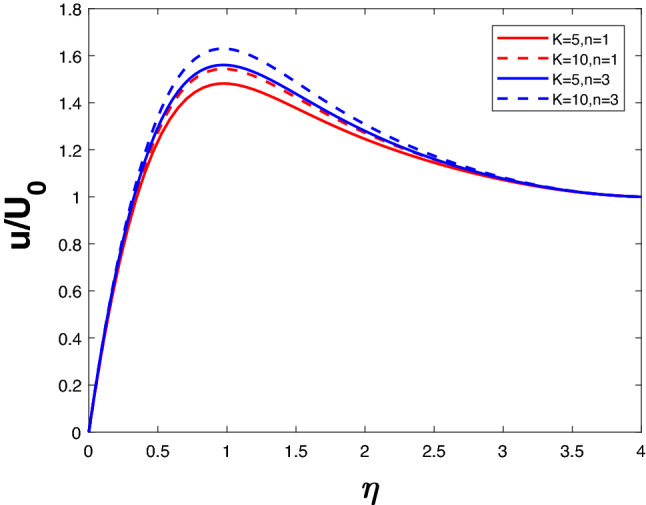
Velocity profile for various *K*.

**Figure 3:**
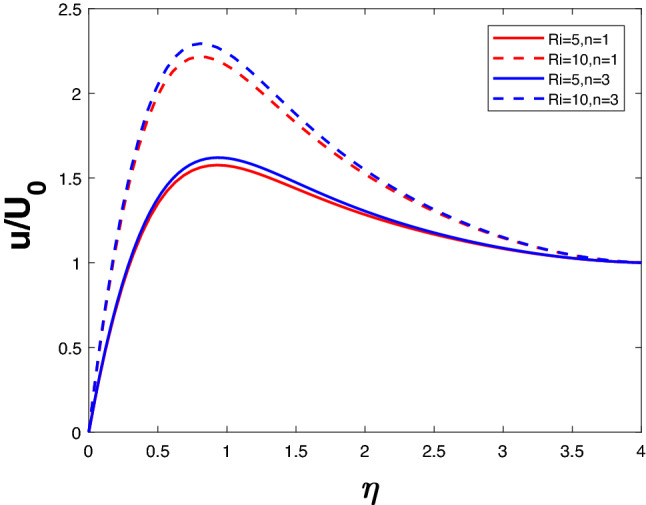
Velocity profile for various *Ri*.

**Figure 4:**
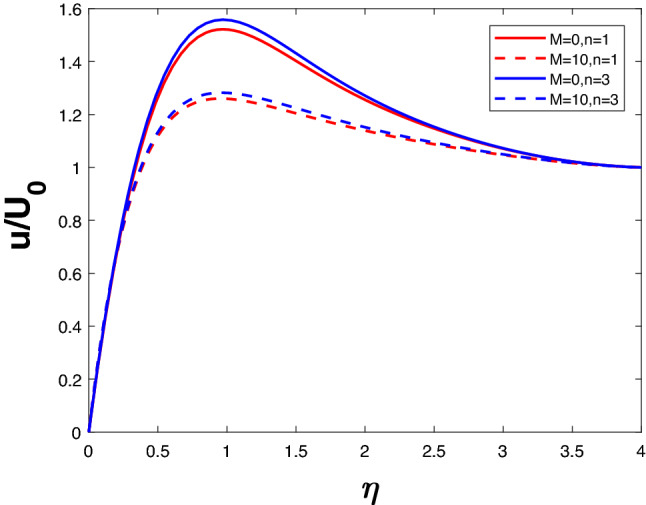
Velocity profile for various *M*.

**Figure 5:**
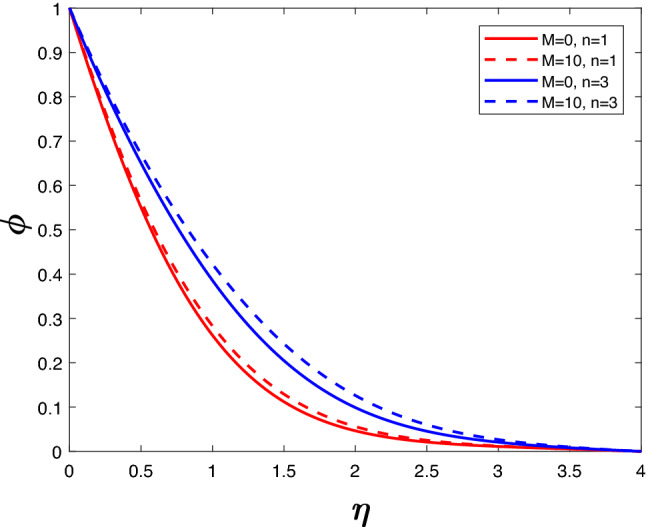
Concentration profile for various *M*.

**Figure 6:**
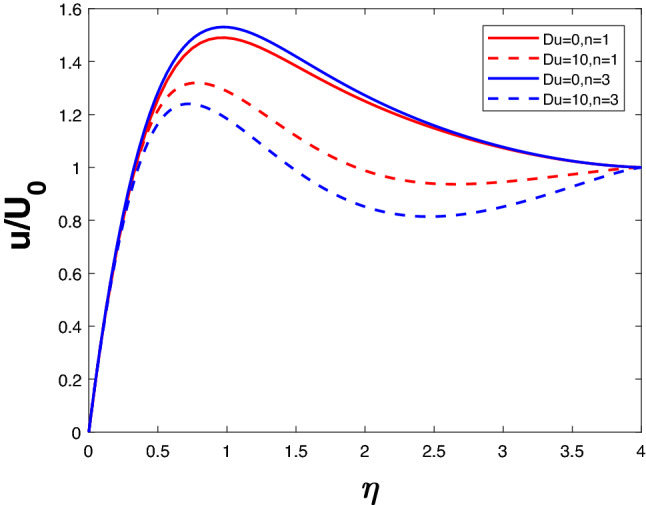
Velocity profile for various *Du*.

**Figure 7:**
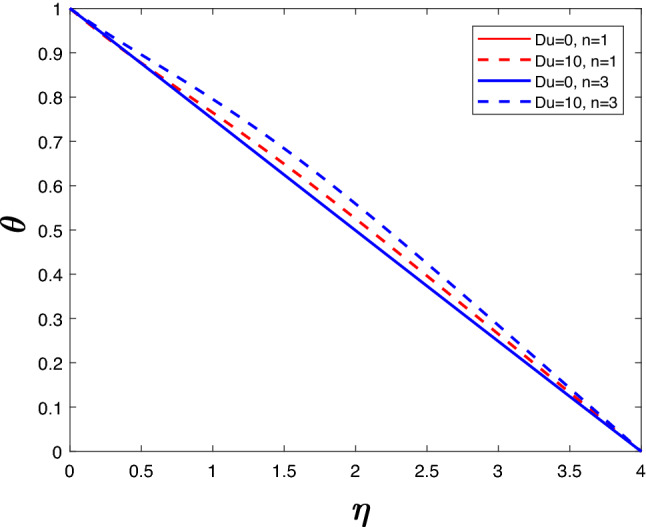
Temperature profile for various *Du*.

**Figure 8:**
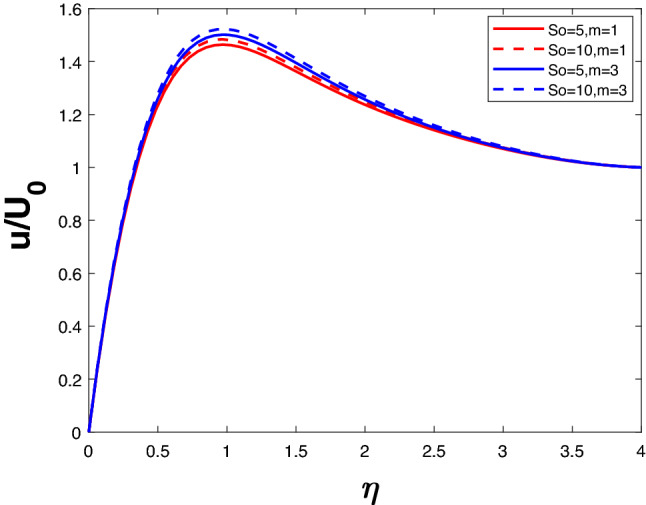
Velocity profile for various *So*.

**Figure 9:**
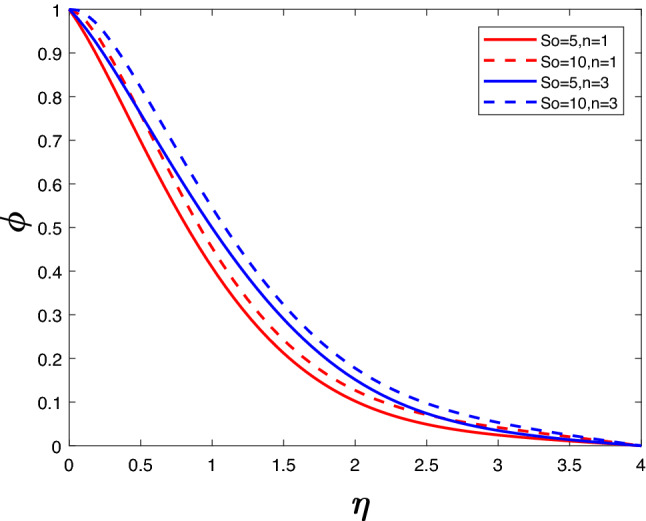
Concentration profile for various *So*.

**Figure 10:**
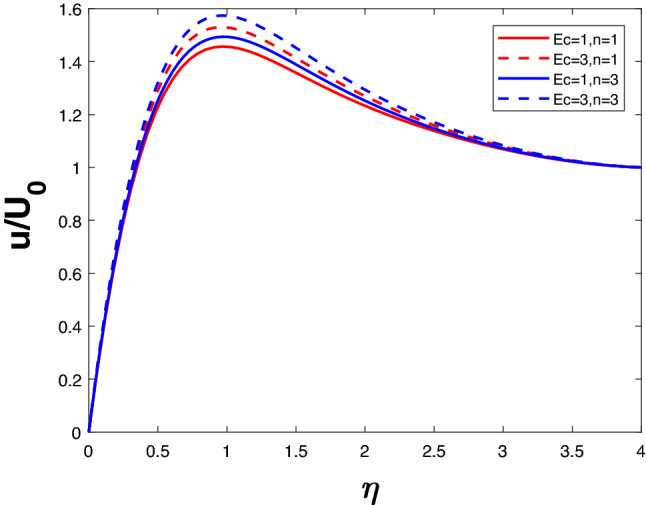
Velocity profile for various *Ec*.

**Figure 11:**
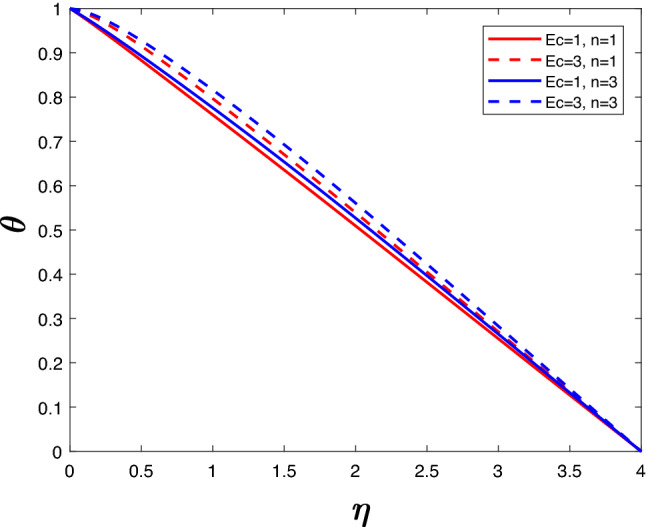
Temperature profile for various *Ec*.

**Figure 12:**
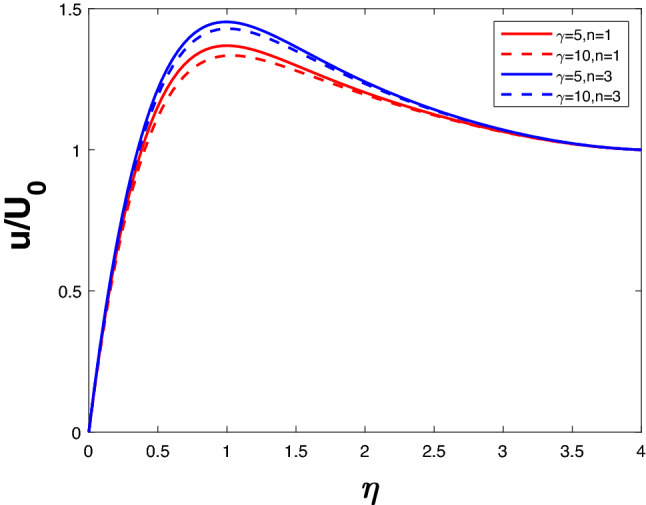
Velocity profile for various $\gamma$.

**Figure 13:**
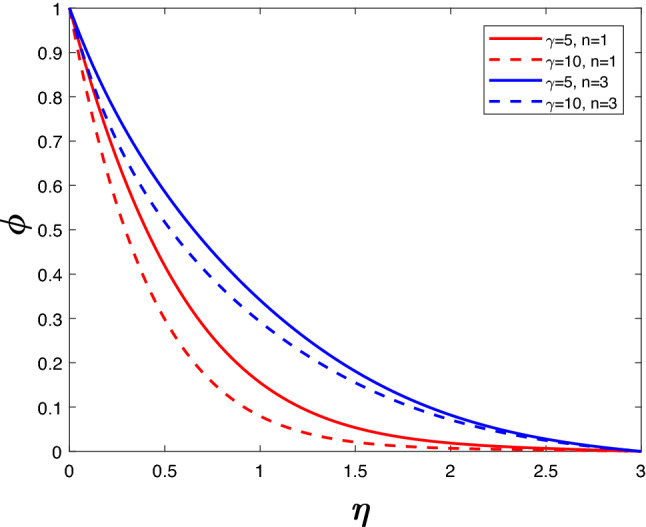
Concentration profile for various $\gamma$.

Specifically, the intention of this work is to analyze how a rotating nanofluid with a constant velocity interacts with a higher order chemical reaction through a plate in conjunction with a porous medium, viscous dissipation and a magnetic field. Keeping in mind, the real-life applications of the order of chemical reaction, the value of *n* is restricted to 3, and a comparison is provided in the following figures for $n\,=\,1$ and $n\,=\,3$. Additionally, the influence of *M*, *Ri*, *Du*, *So*, *Ec*, $\gamma$ on the velocity of the flow, temperature, and concentration of the nanofluid-$\text {Cu}{-}\text {H}_2\text {O}$ are presented by graphs in the Figs. 2, 3, 4, 5, 6, 7, 8, 9, 10, 11, 12 and 13. The nanofluid is formed by suspending copper nanoparticles in water with a volume fraction of 0.15. This problem is solved while considering the thermodynamic properties of both Cu and $\text {H}_2\text {O}$. The thermodynamical properties of Cu and $\text {H}_2\text {O}$ are given in Table 1.

**Table 1:** Thermodynamical properties of nanofluids (Oztop et al.^24^).

Thermodynamical properties	$\text {H}_2\text {O}$	Cu
$C_p$ (J/(kg K))	4179	385
$\rho$ (kg/m$^3$)	997.1	8933
$\kappa$ (W/m K)	0.613	400
$\beta \times 10^{-5}\,(\text {K}^{-1})$	21	1.67

In this paper, for the Figs. 2, 3, 4, 5, 6, 7, 8, 9, 10, 11, 12 and 13, we have used $Ri \,=\, 4; R \,=\, 2; K \,=\, 2; Pr \,=\, 6.785; Du \,=\, 3; Sc \,=\, 0.6; \gamma \,=\, 2; f_w \,=\, 0.5; Ec \,=\, 1; So \,=\, 1; N \,=\, 1;$

Variable parameters are mentioned against the respective graphs.

Figure 2 depicts the velocity for different *Ri* and *n*. For increasing *Ri*, there is an increase in buoyancy due to which the fluid velocity increases. An increase in *n* increases the velocity as well. Figure 3 represents the velocity profile for increasing *n* and *K*. A lower value of *n* results in a lower velocity profile. The velocity increases with an increase in *K* for a given *n* value. Since the ability of the fluid to penetrate into the medium increases with increasing permeability parameter, fluid velocity increases.

The velocity profile in Fig. 4 is illustrated for a variety of *M* and *n* values. It is evident that velocity decreases as *n* increases. Velocity falls when *M* increases for a specific value of *n*. The Lorentz force begins to take control when *M* increases, thus lowering the velocity. Figure 5 represents concentration profile for different *M* and *n*. A higher *n* value results in higher concentration. The concentration increases with an increase in M for a given *n*.

**Table 2:** Comparison of present results with Alam et al.^7^ with $Ri \,=\, 4; N \,=\, 0.5; R \,=\, 0.2; K \,=\, 0.5; M \,=\, 0.5; Pr \,=\, 0.71; Du \,=\, 0.2; Sc \,=\, 0.6; \gamma \,=\, 0; Ec \,=\, 0.01; So \,=\, 0.2; \phi _{0}\,=\,0$.

*Ec*	Alam et al.^7^	Present results
	$f''(0)$	$f''(0)$
0.01	− 3.691740	− 3.686071
0.05	− 3.721830	− 3.719738
0.10	− 3.761275	− 3.763524
0.15	− 3.802958	− 3.809364
0.20	− 3.847133	− 3.857470
0.25	− 3.894094	− 3.908065

**Table 3:** Variation of local skin friction coefficient, Nusselt number, Sherwood number for various values of *Du* for *n* = 1, 2, 3.

*Du*	*n*	$f''(0)$	$- \theta '(0)$	$- \phi (0)$
1	1	− 2.308111	0.216378	0.841627
	2	− 2.355917	0.218110	0.924221
	3	− 2.378565	0.221718	1.079250
2	1	− 2.307127	0.217289	0.842691
	2	− 2.355410	0.220057	0.924967
	3	− 2.378237	0.225729	1.079490
3	1	− 2.306160	0.218143	0.843773
	2	− 2.354914	0.221948	0.925727
	3	− 2.377922	0.229694	1.079731
4	1	− 2.305205	0.218937	0.844875
	2	− 2.354429	0.223782	0.926501
	3	− 2.377616	0.233613	1.079975

**Table 4:** Variation of local skin friction coefficient, Nusselt number, Sherwood number for various values of *So* for *n* = 1, 2, 3.

*So*	*n*	$f''(0)$	$-\theta '(0)$	$-\phi (0)$
1	1	− 2.380070	0.217950	0.827176
	2	− 2.356993	0.221777	0.909210
	3	− 2.305027	0.229576	1.063647
2	1	− 2.377922	0.218143	0.843773
	2	− 2.354914	0.221948	0.925727
	3	− 2.306160	0.229694	1.079731
3	1	− 2.375789	0.218329	0.860279
	2	− 2.352849	0.222114	0.942146
	3	− 2.304310	0.229808	1.095714
4	1	− 2.373669	0.218510	0.876697
	2	− 2.350800	0.222274	0.958470
	3	− 2.302476	0.229918	1.111599

A velocity profile can be observed in Fig. 6 for various values of *Du* and *n*. The increase in velocity is proportionate to *Du*. The velocity is not affected by an increase in *Du* when *n* is low. As *n* rises, the flow velocity increases at higher *Du*. At high *Du* and high *n*, the flow velocity is maximum. It is illustrated in Fig. 7 how the temperature changes as *Du* and *n* increase. Increasing *n* seems to increase the temperature of the system when *Du* is higher. If *Du* is lower, increasing *n* does not increase the velocity of the system. An enhancement in *Du* increases the molecular collisions from hotter to colder regions, which enhances the temperature.

Figures 8 and 9 reveal velocity and concentration profile for different *So*, *n*. For a particular value of *n*, an increase in *So* increases velocity as well as concentration. This is because of the influence of thermal gradients on the diffusing species. Increasing *n* rises the velocity profile and the concentration profile.

Kinesis and heat are measured by Eckert number (viscous dissipation parameter). In the presence of viscous fluid stresses, kinetic energy is changed into internal energy. Higher *Ec* indicates high kinetic energy resulting in increased fluid vibrations and larger collisions between molecules. The boundary layer region becomes hotter as the amount of molecule collisions increases, dissipating more heat. A more rapid flow and higher temperature are therefore associated with a rise in viscous dissipation parameter. With an increase in *n*, collisions will increase, resulting in higher velocity and temperature. Figures 10 and 11 illustrate these trends.

Figures 12 and 13 depict velocity and concentration profiles for increasing values of $\gamma$ and *n*. An increase in $\gamma$ translates into a rise in the number of molecules of solute going through chemical reactions, leading to a reduction in velocity and concentration. Increasing *n* rises the velocity profile and the concentration profile despite the opposing effects of the chemical reaction parameter.

Table 2 provides a limiting case comparison of our results with the results of Alam et al.^7^ with $Ri \,=\, 4; N \,=\, 0.5; R \,=\, 0.2; K \,=\, 0.5; M \,=\, 0.5; Pr \,=\, 0.71; Du \,=\, 0.2; Sc \,=\, 0.6; \gamma \,=\, 0; Ec \,=\, 0.01; So \,=\, 0.2; \phi _{0}\,=\,0$. Our results are in good agreement with the existing literature. Tables 3 and 4 shows values of $f''(0)$, $- \theta '(0)$, $- \phi '(0)$ for increasing *Du*, *So* when $n\,=\, 1, 2, 3$. As seen, as *Du* increases, it increases $-\phi '(0)$ and as *So* increases $- \theta '(0)$ also increase because of Thermo-diffusion and Diffusion-thermo effects created in the fluid.

## Conclusion

A comprehensive investigation is performed with a focus on the influences of higher order chemical reaction, magnetic parameter, permeability parameter, viscous dissipation, Dufour and Soret effects on $\text {Cu}{-}\text {H}_2\text {O}$ nanofluid through a vertical plate. The $\text {Cu}{-}\text {H}_2\text {O}$ nanofluid is considered to flow through a constant velocity acting along with it. This investigation leads us to the following conclusions.

- Heat and mass transfer are more rapid in higher order chemical reaction than in low order reaction.
- Increasing the values of magnetic parameter *M*, chemical reaction parameter $\gamma$ opposes the nanofluid flow while an increase in Dufour number *Du*, permeability paramter *K*, Eckert number *Ec* aids to the flow of the nanofluid.
- The temperature of the nanofluid rises as the heat in boundary layer rises due to a rise in Dufour number *Du* and Eckert number *Ec*.
- The concentration of nanofluid boosts up with an increase in Soret number *So* while an opposite trend is observed for increasing values of chemical reaction paramter $\gamma$.
- For a particular order of a chemical reaction, the rate of heat transfer increases for increasing Soret number *So* while an enhancement in Dufour number *Du* enhances the mass transfer rate.

Moreover, the results of this investigation can be applied to industries manufacturing chemicals such as fertilizers, polymers, and color dyes on a large scale where the faster rates of chemical reactions are significant.

## Data Availability

The datasets used and/or analysed during the current study available from the corresponding author on reasonable request.
